# Podoplanin as an Attractive Target of CAR T Cell Therapy

**DOI:** 10.3390/cells9091971

**Published:** 2020-08-26

**Authors:** Masazumi Waseda, Shin Kaneko

**Affiliations:** Shin Kaneko Laboratory, Department of Cell Growth and Differentiation, Center for iPS Cell Research and Application (CiRA), Kyoto University, Kyoto 606-8507, Japan; m.waseda@cira.kyoto-u.ac.jp

**Keywords:** T cell immunotherapy, tumor-specific antigen, chimeric antigen receptor (CAR), cancer-specific monoclonal antibody (CasMab), induced pluripotent stem (iPS) cell

## Abstract

To date, various kinds of cancer immunotherapy methods have been developed, but T cell immunotherapy is one of the most promising strategies. In general, T cell receptor (TCR) or chimeric antigen receptor (CAR) is used to modify the antigen specificity of T cells. CARs possess an underlying potential with treatment efficacy to treat a broad range of cancer patients compared with TCRs. Although a variety of CAR molecules have been developed so far, the clinical application for solid tumors is limited partly due to its adverse effect known as “on-target off-tumor toxicity”. Therefore, it is very important for CAR T cell therapy to target specific antigens exclusively expressed by malignant cells. Here, we review the application of T cell immunotherapy using specific antigen receptor molecules and discuss the possibility of the clinical application of podoplanin-targeted CAR derived from a cancer-specific monoclonal antibody (CasMab).

## 1. Introduction

In our body, there exist numerous varieties of immune cells, such as T cells, B cells, natural killer (NK) cells, granulocytes, macrophages, and dendritic cells, and they all act to protect us from different kinds of infections and diseases. Utilizing methods to enhance or to activate these immune cells in order to treat cancer is a fundamental strategy that is extensively applied in cancer immunotherapy. To date, various kinds of cancer immunotherapy methods have been developed, but one method that has been extensively applied in research and development is the use of T cells.

T cells are derived from hematopoietic stem cells that differentiate in the thymus. Once migrated to the thymus, T-cell progenitors proliferate in response to interleukin-7 (IL-7) and Delta-like 4 (DLL4), and begin to differentiate into T cells during this process [1,2]. Inside the T-cell progenitors that are stimulated by IL-7 and DLL4, the T cell receptor (TCR) gene locus undergoes rearrangement forming a diverse repertoire; however, appropriate selection within the thymus results in the formation of T cells expressing specific TCRs that are responsible for appropriate responsiveness after being chosen, and then they migrate into the periphery.

Cytotoxic T lymphocytes (CTLs) are the specific T cells that express CD8, and via the TCR they can distinguish between the complexes of antigen peptides and human leukocyte antigens (HLAs) that are present on the cell surface, and thereby possess the function of attacking abnormal cells in an antigen-specific manner. Consequently, it is believed that generating large numbers of CTLs that can specifically recognize cancer cells would enable us to develop effective treatments for specifically targeting the cancer cells. In this review article, we will outline the application of cancer immunotherapy using CTLs with incorporated antigen recognition receptors and explain the possibility of the clinical application of chimeric antigen receptor (CAR) T cell therapies that target the mucin-type glycoprotein, podoplanin (PDPN), and finally the possibility of using CAR T cell therapies that would leverage the new technology of induced pluripotent stem (iPS) cells.

## 2. Antigen Receptor-Transduced CTL Therapy

Tumor infiltrating lymphocytes (TILs) that are specifically responsive to cancer cells were shown to exist in the tumors that were excised from the patients, and since then, several reports were released regarding the possibility to expand TILs ex vivo and use them in cancer treatment [3]. Thereafter, research on TILs has drawn the attention of many researchers. In recent research, it has clearly been shown that the state of differentiation of T cells used for the treatment and their effectiveness for cancer treatment are closely related, thereby increasing the importance of methods that involve selective proliferation of T cells at an early stage of differentiation and introducing genes into them [4]. However, the methods and duration of treatments for each patient differ, and it might be because a sufficient number of T cells cannot be obtained in an early state of differentiation from the patient’s TILs. Consequently, an alternate strategy was devised whereby TCR genes were cloned from cancer cell-specific TILs and transduced into the less or moderately differentiated T cells for the purpose of treatment, and this has been clinically tested.

In many cancer cells, various gene mutations have been shown to accumulate in the genome. Some of these genetic mutations are responsible for expressing specific antigens called “neoantigens”. Interestingly, it has been found that TILs include certain T cells that can specifically recognize these neoantigens [5]. Methods have been developed for determining the neoantigen-specific TCRs by combining various screening systems with sequencing technologies including whole exome sequencing and gene expression analysis of the normal tissue and tumor tissue derived from the same patients [6]. With the advancements in the next-generation sequencing technologies and improvements in the accuracy of antigen prediction algorithms, it is becoming possible to rapidly generate CTLs that are equipped with neoantigen-specific TCRs and can target the specific tumor cells of individual patients.

**Figure 1:**
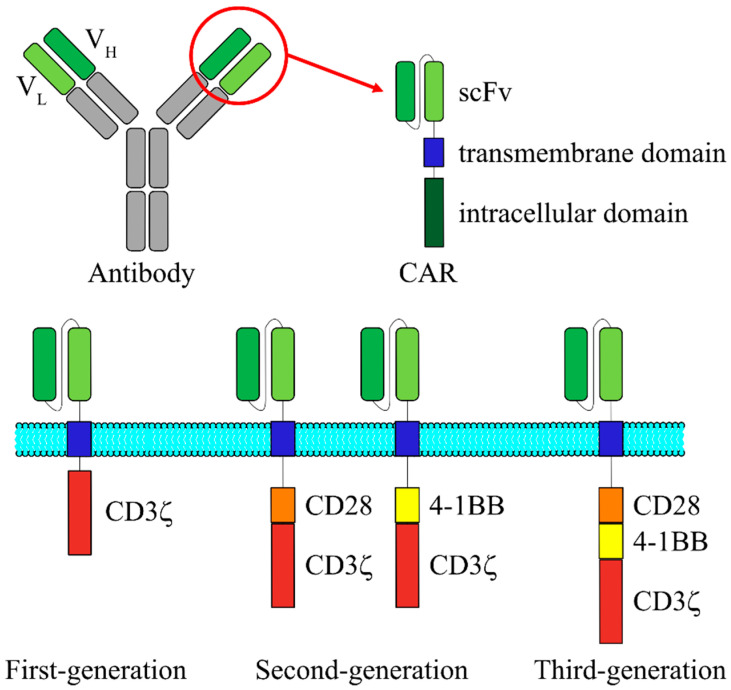
Schematic structure of the three generations of CARs. CAR is a fusion protein composed of an extracellular scFv that is derived from V_H_ and V_L_ regions of an antibody, a transmembrane domain and intracellular signaling domains derived from T cell signaling proteins. First-generation CAR contains only CD3ζ chain for signal transduction. In addition to the CD3ζ chain, second-generation CAR possesses one of the costimulatory receptor-derived signal transduction domains, such as from CD28 and 4-1BB. Third-generation CAR consists of two costimulatory domains. CAR, chimeric antigen receptor; scFv, single chain variable fragment; V_H_, variable heavy chain; V_L_, variable light chain.

Meanwhile, a new antigen recognition receptor called “CAR” has been developed, and this novel receptor is being extensively used for the clinical applications. In general, CAR-expressing T cells are prepared by the following procedures: the desired T cells are harvested and purified from the apheresis product from the patient or an allogeneic donor, followed by T cell activation on anti-CD3/28 beads serving as artificial antigen-presenting cells. The activated T cells are then genetically modified ex vivo by introduction of a gene encoding CAR molecule, and the CAR transduced cells are further expanded ex vivo. When the genetically modified T-cell product has been prepared, the patient receives lymphodepleting preparative regimen followed by adoptive transfer of CAR T cells. CAR is an artificial protein with a basic structure that is comprised of a single chain variable fragment (scFv) derived from an antibody on the N-terminal and a CD3ζ chain on the C-terminal (Figure 1). Between these two terminals, a hinge and a transmembrane domain are also incorporated [7]. First-generation CARs, which do not possess costimulatory receptor-derived signal transduction domain, exhibit high cytotoxic efficacy against target cells in vitro, but exhibit almost no cytotoxic efficacy in clinical trials. This was believed to be due to the poor potential of T cell proliferation after activation, which would further prevent the CAR T cells from acting for longer durations within the patients [8,9]. In contrast, in addition to the composition of first-generation CARs, second-generation CARs are shown to possess one of the costimulatory receptor-derived signal transduction domains, such as from CD28 and 4-1BB, and the CAR T cells did not lose the proliferation capacity even when they were repeatedly stimulated. Therefore, they demonstrated excellent IL-2 release and cell proliferation characteristics after the stimulation [10,11,12]. Third-generation CARs possess two costimulatory receptor-derived signal transduction domains and have been reported to succeed in cytokine release following antigen stimulation [13]. In many different reports using second- and third-generation CARs, it has not been clearly demonstrated: which one is better for CAR T therapy? However, compared to the first-generation CARs, T cells with CARs of the second and third generations have been shown to exhibit better proliferation, cytokine production, and cytotoxic activity, and longer survival in vivo [14,15,16].

Since TCR can target the complexes of HLA and the peptides that are presented, treatment is possible by using only the TCR when the patients possess specific HLAs (HLA restriction). However, it is common to observe a decline or elimination of HLA expression on cancer cells, which is a well-known mechanism whereby cancer cells escape from the immune system [17]. Consequently, CTL therapies using TCR are required to overcome the issue of HLA expression. In contrast, since CARs do not have HLA restriction, they have the advantage of being used for the treatment of any kind of cancer patients as long as the cancer cells can express the target antigen. In addition, for the activation of T cells, it is necessary to have co-signaling via costimulatory receptors in addition to the signaling induced by TCR. Moreover, by using the CARs of the second-generation or later, it is possible to generate both the signals simultaneously with a single CAR, so that they exhibit cytotoxicity against cancer cells that do not express ligands for the costimulatory receptors.

More recent studies have suggested the potential application of CAR T cell therapy for other diseases. For example, Aghajanian et al. reported the efficacy of CAR T cell therapy for this disease using a mouse model of myocardial fibrosis [18]. Fibrosis is a condition that is observed in conjunction with numerous myocardial diseases, and activated fibroblasts produce excessive extracellular matrix proteins. However, while there is now a therapeutic method that directly targets fibrosis, it has limitations, and new treatment methods are needed. Therefore, to discover new therapeutic targets, gene expression analysis was performed in cardiac tissues obtained from heart transplant donors and recipients using an RNA sequence analysis database; fibroblast activation protein (FAP) was identified as a target candidate as a result. FAP is a glycoprotein that is expressed in fibrotic tissues and various cancer cells but is rarely expressed in normal human and mouse tissues. FAP has also been discussed in reports from studies where it is used as a target for cancer treatments using CAR T cells [19,20,21]. As a result of human and murine cardiac tissue staining, FAP was found to be strongly expressed in fibroblasts present in myocardial fibrotic tissue. Therefore, when CAR T cells targeting FAP were transplanted into myocardial fibrosis model mice, fibrosis was significantly suppressed as compared with the non-treated group, and the cardiac function was equivalent to that of normal tissue.

In this way, CARs possess an underlying potential with treatment efficacy to treat a broad range of patients not only with cancer but also with other diseases compared to TCRs.

## 3. Extending CAR T Cell Therapy from B Cell Malignancies to Solid Tumors

The clinical trials that have currently progressed extensively are performed for the CAR T cell therapies that target hematologic malignancies with B cells that express CD19. CD19 is expressed in tumor cells, such as in non-Hodgkin’s lymphoma, acute lymphoblastic leukemia (ALL), and chronic lymphocytic leukemia (CLL), but is not expressed in hematopoietic stem cells, non-B-cell hematopoietic cells, and non-lymphoid tissues. Previously, a high rate of complete remission has been obtained in phase I and II clinical trials, targeting the children or young patients with relapsing and intractable CD19-positive ALL. Additionally, there have been reports of cases where remission was maintained for long periods, such as over 2 years since the treatment [22]. Confirming its considerable clinical efficacy, treatment with CAR T cells that target this molecule was approved by the U.S. Food and Drug Administration (FDA) in 2017, and the development of CAR T cell treatments that target hematological malignancies is being pursued actively worldwide.

However, previous studies have suggested that there are at least two patterns in which CAR T cell therapies are ineffective [22]. The first involves instances where transplanted CAR T cells do not actively proliferate and thus are unable to exert a sufficient therapeutic effect. For example, it is believed that T cells, which are the source of CAR T cells, can become exhausted by treatments patients previously received, such as radiotherapies and anticancer chemotherapy. Therefore, in TCR-introduced T cell therapy, it is important to select T cells in an appropriate state to generate CAR T cells. The second is when cancer cells evade CAR T cell attack by mutation or loss of expression of the CD19 antigen epitope of CAR T cells [23]. For example, in a Phase II clinical trial investigating pediatric and adolescent patients with relapsed or refractory B-cell acute lymphoblastic leukemia (B-ALL), only one patient bearing CD19-positive leukemia cells relapsed, while fifteen cases of CD19-negative recurrence were confirmed, which is an important issue in achieving complete remission of the disease [24]. Studies of CAR T cell therapies targeting multiple antigens are currently ongoing as part of a strategy to overcome this issue. To date, 4-1BB-based second-generation CAR T cells targeting antigens such as CD20, CD22, CD123, in addition to CD19, have been evaluated using a xenograft model. In these studies, CAR T cells targeting two different antigens have been shown to significantly suppress recurrence due to CD19-negative cells in comparison to CAR T cells targeting CD19 alone [25,26,27]. Further, it has been reported as a result of clinical studies that the administration of CAR T cells targeting CD19 and CD22 can improve the therapeutic effect [28]. In addition, by combining fluorescein isothiocyanate (FITC)-labeled antibodies and anti-FITC CAR proteins, a method for addressing multiple target molecules using a single CAR has been developed, which enables simplified targeting of multiple antigens [29]. As such, CAR T cell therapies targeting multiple antigens are expected to become mainstream in the future to prevent the recurrence of tumor cells that have lost antigenicity.

However, in contrast to the clear clinical efficacy of CAR T cells against the hematological malignancies, the clinical efficacy of CAR T cell therapies against solid tumors has not been established yet. Major factors contributing to this are the need to overcome the mechanisms of immunosuppression in the tumor microenvironment and the requirement of accumulating the CAR T cells at the tumor site. To date, immunotherapy involving IL-2 administration has been widely used to enhance the antitumor effect, and a certain level of efficacy has been observed in metastatic melanoma and renal cell carcinoma patients [30]. However, previous studies have shown that IL-2 is involved in activation-induced cell death, suppression of memory T cell proliferation and survival, and the proliferation of regulatory T cells, and it has also been shown that there is a risk of death associated with further treatment related to high-concentration IL-2 administration [31,32,33,34]. As such, a new T cell activator capable of avoiding these issues is required, and IL-15 is currently considered to be one of the most promising candidates. Similar to IL-2, IL-15 uses a complex containing CD122 and CD132 as a receptor and it is known to show similar properties than IL-2, such as the ability to induce T cell proliferation. Meanwhile, various types of IL-15, such as secretory IL-15, secretory IL-15/IL-15R complex, and membrane-bound IL-15, have been studied. IL-15 has been reported to maintain memory T cells and suppress apoptosis [35,36,37]. Furthermore, co-expression of IL-15 in CAR T cells has been confirmed to markedly improve the antitumor effect in xenograft model mice [38,39]. In addition, there was also a report that the generation of CAR T cells that simultaneously express IL-7, which is known to promote T cell proliferation and survival, and CCL19, which is a chemoattractant of T cells and dendritic cells, was successful in obtaining an extremely high antitumor effect [40]. As described above, studies using various humoral factors other than IL-2 are ongoing worldwide, and there have been numerous attempts to find practical applications for CAR T cells capable of remarkable antitumor effects against solid tumors. An additional issue is that solid tumors are highly heterogeneous, which makes it difficult to identify a suitable, single target molecule [41].

## 4. Adverse Effects and Toxicities of CAR T Cell Therapy

The most prominent toxicities of CAR T cells are cytokine release syndrome (CRS) and central nervous system (CNS) complications. CRS is thought to be caused by cytokines secreted by administered T cells, and a series of symptoms including fever of over 39 °C and hypotension were observed mainly from several days to 1–2 weeks after administration [42,43]. However, neurological toxicities may occur at a different time or independently of CRS, and it is speculated that this may be due to a mechanism different from CRS in at least some cases. As mild to moderate neurological side effects, various symptoms such as headache, mild aphasia, movement disorder, encephalopathy, and delirium have been reported, but in severe cases, the occurrence of serious symptoms such as coma, intracranial hemorrhage, and brain edema was reported [44,45,46].

Although many mechanisms have not yet been fully characterized, our pathophysiological understanding of CRS and CNS complications continues to grow. For example, recent findings from a study of the murine xenograft model suggest that not only CAR T cells but also monocytes/macrophages play important roles in the development of CRS and neurological side effects [47]. Specifically, in humanized mice with a high leukemia burden, the main source of IL-1 and IL-6 during CRS development was human-derived monocytes. Although the inhibition of IL-6 receptors with tocilizumab prevented the onset of CRS, CNS complications were still confirmed. By contrast, the inhibition of IL-1 signal transduction using anakinra, an IL-1 receptor antagonist, was able to suppress both CRS and CNS complications. As IL-1 secretion precedes IL-6 production, and IL-1 induces IL-6 secretion, IL-1 is believed to facilitate the release of IL-6 into the bloodstream in the patients with CRS. The excessive release of cytokines into the bloodstream by the activation of CAR T cells and monocytes/macrophages stimulates endothelial cells exposed to high concentrations of IL-6 and IFN-γ [48,49]. When vascular endothelial cells throughout the body are activated and vascular permeability is enhanced, blood pressure falls and hypoalbuminemia is induced, resulting in systemic capillary leakage syndrome. In vitro experiments have also shown that IL-6 disrupts the blood–brain barrier (BBB)—by acting on vascular endothelial cells and promoting a decrease in the expression of tight junction-related molecules [50]. In vitro findings at the BBB are supported by results from studies of patients who developed fatal cerebral edema and had severe CRS and CNS complications [44,51]. Based on these findings, the development of novel therapeutic agents that are able to suppress the enhancement of vascular permeability and BBB breakdown, as well as prevent CRS and CNS complications, can be expected to make further advancements.

Although no optimal treatment for CRS has been established, previous studies have shown that anti-IL-6 receptor antibody therapy and corticosteroid administration show specific effects. CRS has been observed to be associated with the elevation of IL-6 in the early stage of clinical trials using CAR T cells, which has motivated researchers to try tocilizumab, an inhibitory antibody targeting the IL-6 receptor [52]. Currently, tocilizumab is the standard treatment for CRS and was approved by the FDA in 2017. So far, no adverse events have been reported with tocilizumab administration for CRS. There was also a concern that the antitumor activity of CAR T cells may be inhibited by the early administration of tocilizumab, but there is currently no evidence supporting this claim. The main limitation of tocilizumab is its inability to cross the BBB. Accordingly, CNS complications have been reported to still develop even after recovery from CRS by tocilizumab administration [24,53]. In fact, blood levels of IL-6 become elevated even after the IL-6 receptor is blocked by tocilizumab, and the brain will continue to be exposed to high levels of IL-6. Therefore, a method of directly inhibiting IL-6 by administering the anti-IL-6 antibody siltuximab to suppress CNS complications has also been investigated. Moreover, considering that it has a broad anti-inflammatory effect, corticosteroids are also an option in the treatment of CRS. However, given that corticosteroids are potentially T cell cytotoxic and this may affect their therapeutic efficacy, corticosteroids should be reserved for cases that are non-responsive to tocilizumab. Steroids are also frequently used to reduce CNS complications in cases that are non-responsive to tocilizumab monotherapy. Dexamethasone is also a strong candidate, considering its ability to reach the CNS.

Another serious issue with CAR T cell therapies is on-target off-tumor toxicity. This is because CAR T cells exhibit toxicity towards normal tissues expressing even small amounts of the target molecule. For example, there have been reports of respiratory distress and pulmonary infiltration, which were observed following the administration of ErbB2-specific CAR T cells, and consequently, the patients died 5 days after treatment. This is believed to be due to the low-level expression of ErbB2 on lung epithelial cells [54]. Furthermore, delayed-onset respiratory toxicity has been confirmed in clinical trials using CAR T cells targeting carcinoembryonic antigen related cell adhesion molecule 5 (CEACAM5), and on-target off-tumor toxicity has become a concern when tumor-associated antigens are targeted [55]. In another case, unexpected hepatotoxicity was also caused in a Phase I study targeting carbonic anhydrase IX (CAIX) expressed in renal cell carcinoma [56]. Although not seen in preclinical studies, normal bile duct epithelial cells are believed to express low levels of the CAIX antigen and are injured by CAR T cells. In addition, while clinical studies with CAR T cells targeting common antigens such as mesothelin, carcinoembryonic antigen (CEA), and ganglioside (GD2) have been conducted in the past, no remarkable toxicity was reported. However, virtually no antitumor activity was observed in these clinical studies, and it is highly probable that toxicity did not occur due to insufficient CAR T cell activity.

Various techniques have been developed to remove administered CAR T cells to address severe toxicity for instances unable to be controlled through the aforementioned treatment methods. One technique involves the expression of target antigenic molecules such as epidermal growth factor receptor (EGFR) and CD20 in CAR T cells and removal of CAR T cells administered using already clinically approved antibodies such as cetuximab and rituximab [57,58]. Another technique involves incorporating a “suicide gene” into CAR T cells. The system using inducible caspase-9 (iCasp9) produces a particularly rapid response, and its efficacy has been confirmed through clinical trials. iCasp9 is immediately dimerized by the administration of low molecular weight compounds, and it is possible to rapidly remove cells by activating the apoptotic pathway [59,60]. However, as these techniques have a substantial impact on antitumor efficacy, they are believed to be useful as a safety contingency in the case of lethal toxicity caused by CAR T cells that cannot be controlled by other methods. A recent report has stated that this activity can be regulated while maintaining CAR T cells in the body by using dasatinib, which inhibits multiple tyrosine kinases, but it will take time for a practical application to be developed [61]. Consequently, it is extremely important in CAR T cell therapies that only the antigens that are specifically expressed in cancer cells are applied.

## 5. PDPN and Anti-PDPN CasMab

Human podoplanin (PDPN) has a total length of 162 amino acids and consists of a highly glycosylated extracellular domain, a single-pass transmembrane domain, and a short intracellular domain of nine amino acids. PDPN itself is not known to have any enzymatic activity; however, it interacts with various other proteins, including C-type lectin-like receptor-2 (CLEC-2), CD44, galectin 8, C-C chemokine ligand 21 (CCL21), ezrin, moesin, protein kinase A (PKA), and cyclin dependent kinase 5 (CDK5), and has been reported to possess various other functions [62,63]. For example, PDPN colocalizes with the ezrin, radixin, and moesin (ERM) family proteins, and binds directly to ezrin and moesin. In addition, overexpression of PDPN leads to phosphorylation of ERM proteins and activation of RhoA, thereby increasing motility and induction of epithelial–mesenchymal transition (EMT) [64,65,66].

So far, research about PDPN has been carried out extensively, particularly in the field of cancer. PDPN acts as a molecular marker specific for lymphatic vessels and, because there is a correlation shown to exist between the formation of lymphatic vessels and poor cancer prognosis, it is also frequently used as a diagnostic marker for cancer [67,68]. In addition, increased expression of PDPN has been reported in numerous cases of solid tumors, including squamous cell carcinomas of the lungs, head and neck [69,70], malignant mesothelioma [71,72], and brain tumors [73]. PDPN is often identified to express at the leading edge of a tumor, suggesting that it contributes to metastasis and infiltration of the cancer cells [65]. Moreover, aggregates of circulating cancer cells with platelets through the binding of PDPN to CLEC-2 have been reported to further promote the process of hematogenous metastasis [74]. Therefore, inhibiting the interaction between PDPN and CLEC-2 is believed to be a unique, and feasible treatment strategy for targeting metastasis of the cancer cells [75].

NZ-1 is an anti-PDPN antibody and, in the research established by Kato et al., the authors used the antibodies derived from NZ-1 and demonstrated an anti-tumor effect using the xenograft models of glioma, mesothelioma, lung cancer, and others [76,77]. In addition, treatment with CAR T cells using the third-generation CARs that were designed based on the NZ-1 antibody also exhibited prolonged duration of survival using the xenograft models of glioblastomas [78]. This way, the efficacy demonstrated by treatments that target PDPN has been verified in both in vitro experiments and in xenograft models.

As stated above, the issue that requires most consideration for the clinical application of CAR T cell therapies is on-target off-tumor toxicity. In other words, for this treatment method, it is extremely important to select molecules specifically expressed in cancer cells. PDPN is highly expressed in various intractable tumors, such as lung cancer, malignant mesothelioma, malignant brain tumors, and esophageal cancer; however, it is also known to be expressed in normal cells, including lymphatic endothelial cells, alveolar epithelial cells, skin basal cells, and renal epithelial cells. Consequently, it is difficult to clinically apply CAR T cell therapies that are designed based on the anti-PDPN antibodies, such as NZ-1. However, in the study conducted by Kato and Kaneko, the authors succeeded in developing a cancer-specific antibody, which is known as the cancer-specific monoclonal antibody (CasMab), thereby adding a sense of feasibility to the actual use of CAR T cell therapies that can specifically target PDPN [79]. They focused on the PDPN molecule that is specifically expressed in the human glioblastoma cell line LN229, which had a different glycosylation pattern compared to the normal cells, and thereafter established CasMab by transplanting LN229 cells expressing PDPN (LN229/hPDPN) into mice. Among the established clones, a CasMab clone, LpMab-2, recognized human PDPN-expressing CHO cells (CHO/hPDPN), *N*-glycan deficient CHO/hPDPN cells, and LN229/hPDPN cells, whereas the clone did not recognize sialic acid-deficient CHO/hPDPN cells and *O*-glycan deficient CHO/hPDPN cells. These results indicate that the epitope recognized by LpMab-2 includes sialylated *O*-glycans. Furthermore, the authors determined the LpMab-2 target sequence by using deletion and point mutants of PDPN. As a result of these experiments, they concluded that the important epitope for LpMab-2 is the glycopeptide Thr55-Leu64. CasMab is an antibody that recognizes both the cancer-specific glycosylation and the PDPN-derived peptide sequence. Although it reacts with PDPN expressed by the cancer cells, it has been shown to be unreactive to PDPN expressed by the normal cells. Consequently, these CasMab and their scFv are promising and can be considered for application in PDPN-targeted therapy.

## 6. Conclusions and Perspectives

As discussed in this review article, if one can prepare CARs that maintain CasMab’s antigen specificity, it might be used to prevent the major issue of CAR T cell therapy, i.e., on-target off-tumor toxicity. In that case, this technology will change PDPN into an attractive target for CAR T cell therapy. There are, however, some concerns that should be considered when applying the technology to clinical practice. Although Kato and Kaneko have previously demonstrated that CasMab is a specific antibody directed against aberrantly glycosylated PDPN expressed in some human cancer cell lines and some patient-derived tumors, the number of patients’ samples is very limited. Therefore, more extensive studies are required to further confirm the antigen specificity of CasMab and the applicability of this concept to glioblastoma and other types of cancer. Another concern is the implications of tumor heterogeneity. Since CasMab recognizes both specific glycosylation and a PDPN-derived peptide as described above, it is assumed that the effectiveness of CasMab-based therapies is affected not only by the expression level of PDPN but also by the glycosylation pattern of PDPN. Therefore, a strategy targeting multiple antigens, including cancer-specific glycosylation patterns, may be useful to improve the therapeutic effect.

**Figure 2:**
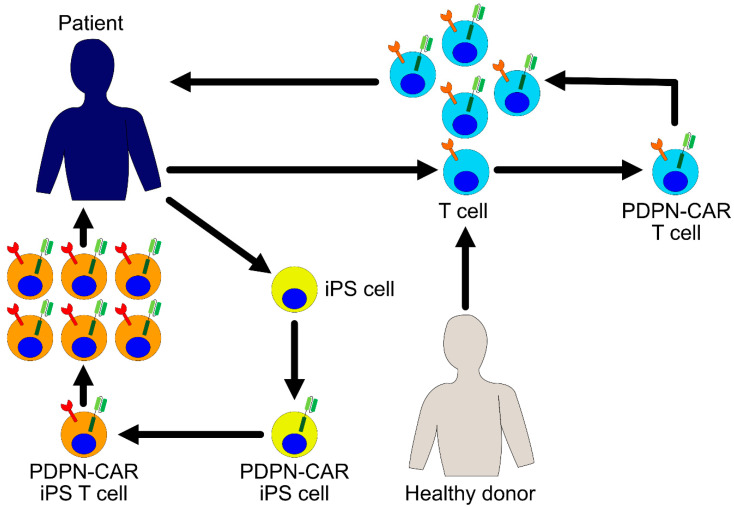
PDPN-CAR expressing T cells for cancer immunotherapy. Patient derived- or healthy donor derived-T cells are harvested and transduced with a PDPN-CAR encoding gene ex vivo. The PDPN-CAR T cells are further expanded ex vivo and infused into the patient. Another possible approach is to use induced pluripotent stem (iPS) cells as a source of T cells. iPS cells are established from patient’s somatic cells and transduced with a PDPN-CAR ex vivo. PDPN-CAR expressing T cells are differentiated from these iPS cells, expanded, and infused into the patient.

In the research studies that have been conducted so far, it has been found that long-term exposure to antigen stimulation and inflammatory signals in chronic diseases, such as cancer and viral infection, causes T cells to enter a state of exhaustion [80]. Exhausted T cells start expressing various inhibitory receptors and gradually lose their proliferation, cytokine production, and cytotoxic potential. Therefore, when the autologous T cells are used in the treatment, there is a concern that even while using the T cells harboring CARs, the proportion of exhausted T cells might affect the efficacy of the treatment. One approach for resolving this issue is developing immunity by renewing treatments that are focused on iPS cell techniques. Previously, we and other groups succeeded in inducing CTL differentiation using the iPS cells, and these regenerated CTLs have been found to exhibit considerable proliferation and cytokine production potential [81,82,83]. Moreover, iPS cells are efficiently transduced by recombinant lentiviral vectors and it is easy to control the quality of the gene-modified iPS cells. In the future, it is possible that the regenerated T cells derived from the iPS cells will become a platform for the expression of CARs and will become an important focus of research. The integration of this research is promising in asserting the development of PDPN-targeting CAR T cell therapies with considerable clinical efficacy for future studies (Figure 2).
